# Supplementary material for: Pirating conserved phage mechanisms promotes promiscuous staphylococcal pathogenicity island transfer
Source: eLife. 2017 Aug 8;6:e26487. doi: 10.7554/eLife.26487 (PMC5779228; doi:10.7554/eLife.26487)
Supplement: Supplementary file 1. [file elife-26487-supp1.docx]

| **Supplementary file 1. Crystallographic statistics of the ϕO11-dUPNPP structure** | | |
| --- | --- | --- |
| **Processed data** | | |
| Beamline | XALOC (ALBA) |  |
| Wavelength (Å) | 0.98 |  |
| Space group | P2_1_ |  |
| Cell dimensions  *a, b, c* (Å)  *α, β, γ* (°) | 33.47, 88.84, 54.15  90, 91.68, 90 |  |
| Resolution (Å) | 54.13–2.1 (2.21-2.1) |  |
| R_merge_ (%) | 0.13 (0.31) |  |
| Mean I/δ(I) | 6.4 (3.7) |  |
| Unique reflections | 18124 (2620) |  |
| Completeness (%) | 98.0 (98.1) |  |
| Redundancy | 3.0 (3.0) |  |
| **Refined data** |  |  |
| R_factor_ (%) | 0.19 (0.20) |  |
| R_free_ (%) | 0.24 (0.28) |  |
| No. non-hydrogen atoms  Protein  Ligand  Water  Mg | 2373  56  74  4 |  |
| RMSD  Bond deviation (Å)  Angle deviation (º) | 0.0077  1.138 |  |
| Average B-factor (Å^2^)  Protein  Ligand  Water  Mg | 33.31  23.37  32.86  20.23 |  |
| Ramachandran Map  Favoured (%)  Allowed (%)  Outliers (%)  PDB accession code | 97.03  2.31  0.66  5MIL |  |

Values in parentheses correspond to the data for the highest resolution shell

R_merge_ = Σ (I -<I>) / Σ<I>

R_factor_= Σ‖Fo|−|Fc‖/Σ|Fo|

R_free_ is the R_factor_ calculated with 5% of the total unique reflections chosen randomly and omitted from refinement
