# Supplementary material for: Pirating conserved phage mechanisms promotes promiscuous staphylococcal pathogenicity island transfer
Source: eLife. 2017 Aug 8;6:e26487. doi: 10.7554/eLife.26487 (PMC5779228; doi:10.7554/eLife.26487)
Supplement: Supplementary file 2. [file elife-26487-supp2.docx]

**Supplementary file 2. Templates and confidence values in SaPIbov1 Stl repressor models generated by I-Tasser^a^ and Phyre2^b^ servers.**

| **Server** | **Template information** | **PDB code** | **Aligned**  **Residues^e^** | **Confidence^f^**  **(%)** | **Identity**  **(%)** |
| --- | --- | --- | --- | --- | --- |
| Phyre2 | PrgX transcription factor (*E. Coli*) | 2GRM | 14-261 | 99.8 | 12 |
|  | Lmo0325 transcription factor (*L. monocytogenes*) | 4RYK | 13-260 | 99.8 | 12 |
|  | Rgg2 transcription factor (*S. dysgalactiae*) | 4YV9 | 14-263 | 99.7 | 12 |
|  | PlcR transcription factor (*B. thuringiensis*) | 2QFC | 12-261 | 99.7 | 13 |
|  | HTH-like transcriptional regulator YbaQ (*E. coli*) | 2EBY | 11-96 | 99.6 | 15 |

| **Server** | **Top threading templates** | **Closest structural analog** | **Top final model** | | |
| --- | --- | --- | --- | --- | --- |
|  |  |  | **C-score^c^** | **TM-score^d^** | **RMSD (Å)** |
| I-Tasser | 2QFC, 5D50, 3U3W, 2Bnn, 4RYK, 2AWI | 2QFC. PlcR transcription factor (*B. thuringiensis*) | -1.65 | 0.51 ± 0.15 | 9.7 ± 4.6 |

^a^See reference (Yang et al., 2015) for details.

^b^See reference (Kelley, Mezulis, Yates, Wass, & Sternberg, 2015) for details.

^c^C-score is a confidence score for estimating the quality of predicted models , typically ranging between -5 and 2, where a higher value signifies a model with a higher confidence.

^d^TM-score is a scale for measuring the structural similarity between two structures. A TM-score >0.5 indicates a model of correct topology and a TM-score <0.17 means a random similarity.

^e^Aligned residues. Part of protein sequence that is aligned with the template sequence.

^f^Confidence represents the probability (from 0 to 100) that the match between the query sequence and the corresponding template is a true homology. A match with confidence >90%, generally should indicate that the query sequence adopts the overall fold shown by the template and that the core of the protein is modelled at high accuracy.
