# Supplementary material for: Pirating conserved phage mechanisms promotes promiscuous staphylococcal pathogenicity island transfer
Source: eLife. 2017 Aug 8;6:e26487. doi: 10.7554/eLife.26487 (PMC5779228; doi:10.7554/eLife.26487)
Supplement: Supplementary file 3. [file elife-26487-supp3.docx]

**Supplementary file 3. SSAPs present in *S. aureus* phages.**

| **SSAP family** | **Bacteriophage** | **Accession number** |
| --- | --- | --- |
| Sak | 80α | YP_001285330 |
|  | φX2 | YP_240871 |
|  | φ53 | YP_239694 |
|  | φ85 | YP_239777 |
|  | φ88 | YP_240716 |
|  | φMR25 | YP_001949813 |
|  | φP954 | YP_003169647 |
| Sak4 | φ52A | YP_240651 |
|  | φ11 | NP_803265 |
|  | φ80 | YP_009268656 |
|  | φ96 | YP_240275 |
|  | φETA | NP_510911 |
| Erf | φSLT | NP_075480 |
|  | φED133 | ADI98419 |
|  | φETA3 | YP_001004345 |
|  | φStB12 | YP_009130692 |
|  | φ5967PVL | YP_007237141 |
|  | φSA97 | YP_009218463 |
|  | φ7247PVL | BAL03577 |
| Redβ | φN315  phiBU01 | NP_835535  YP_009113134 |
