# Supplementary material for: Pirating conserved phage mechanisms promotes promiscuous staphylococcal pathogenicity island transfer
Source: eLife. 2017 Aug 8;6:e26487. doi: 10.7554/eLife.26487 (PMC5779228; doi:10.7554/eLife.26487)
Supplement: Supplementary file 4. — (B) Templates and confidence values in recombinase models generated by Phyre2 servera. [file elife-26487-supp4.docx]

**Supplementary file 4A. Templates and confidence values in recombinase models generated by I-Tasser server^a^.**

| **Protein** | **Top threading templates** | **Closest structural analog** | **Top final model** | | |
| --- | --- | --- | --- | --- | --- |
|  |  |  | **C-score^b^** | **TM-score^c^** | **RMSD (Å)** |
| 80α | 1H2I, 1KN0, 4C2M, 4CO6, 3J16, 2LYX | 1H2I. Rad52 protein (*H. sapiens*) | -2.75 | 0.40 ± 0.13 | 11.8 ± 4.5 |
| 52A | 3DVL, 1Q57, 3BGW, 3HR8, 2CVF, 1CR1. | 3CMT. RecA (*E. coli*) | -1.51 | 0.53 ± 0.15 | 9.3 ± 4.6 |
| SLT | 5CWO, 2NCJ, 4CO6, 3ZEY, 5ª63, 1YKD, 4TQL, 1ZAU, 2E52 | 5CWO. *De novo* designed helical repeat protein | -3.37 | 0.34 ± 0.11 | 13.5 ± 4.0 |
| N315 | 4ROC, 4D7S, 5A9Q, 3IIX, 4NDJ, 4RGL, 1LDD, 1K8T, 1KEK | 4ROC. Transcription factor IIIB (*H. sapiens*) | -3.84 | 0.30 ± 0.10 | 15.7 ± 3.2 |

^a^See reference (Yang et al., 2015) for details.

^b^C-score is a confidence score for estimating the quality of predicted models , typically ranging between -5 and 2, where a higher value signifies a model with a higher confidence.

^c^TM-score is a scale for measuring the structural similarity between two structures. A TM-score >0.5 indicates a model of correct topology and a TM-score <0.17 means a random similarity.

**Supplementary file 4B. Templates and confidence values in recombinase models generated by Phyre2 server^a^.**

| **Protein** | **Template information** | **PDB code** | **Aligned**  **Residues^a^** | **Confidence^b^**  **(%)** | **Identity**  **(%)** |
| --- | --- | --- | --- | --- | --- |
| 80α | Human Rad52 (N-terminal domain) | 1H2I | 70-131 | 93.5 | 26 |
|  | Human Rad52 | 1KN0 | 71-129 | 65.4 | 20 |
|  | Alpha-D-ribose 1-methylphosphonate 5-phosphate C-P lyase (*E. Coli*) | 4XB6 | 111-179 | 59.1 | 21 |
|  | Mitoribosomal protein ML42 (*Sus scorfa*) | 4V1A | 66-94 | 38.4 | 15 |
|  | Perisplasmic protein YceI (*E. coli*) | 1Y0G | 39-91 | 32.6 | 15 |
| 52A | RecA (*E. coli*) | 2REC | 31-161 | 98.5 | 15 |
|  | RecA (*M. smegmatis*) | 2ZRO | 31-161 | 98.5 | 15 |
|  | RecA (*D. radiodurans*) | 1XP8 | 31-161 | 98.5 | 17 |
|  | Rad51 (*S. cerevisiae*) | 3LDA | 31-161 | 98.3 | 15 |
|  | DnaC helicase (*G. kaustophilus*) | 2VYE | 31-245 | 97.7 | 18 |
|  | Adenilato kinase (*P. falciparum*) | 3TLX | 9-45 | 95.9 | 26 |
| SLT | RNase H3 (*Thermovibrio ammonificans*) | 4PY5 | 170-212 | 41.8 | 12 |
|  | Hypothetical protein TT1679 (*Thermus thermophilus*) | 1V8D | 36-126 | 39.6 | 12 |
|  | RNase H-like (*Thermococcus kodakarensis)* | 1IO2 | 170-212 | 32.9 | 12 |
|  | RNase H3 (*Aquifex aeolicus*) | 3VN5 | 170-212 | 32.4 | 10 |
|  | Transcription factor E2F1 (*H. sapiens*) | 2AZE | 6-38 | 17.8 | 14 |
| N315 | GFP-like protein (*Pontellina plumata*) | 2G3O | 116-179 | 50.4 | 14 |
|  | Penicillin V acylase (*Lysinibacillus sphaericus*) | 2PVA | 24-81 | 25.4 | 12 |
|  | Calnexin chaperone (*Canis lupus*) | 1JHN | 91-138 | 22.4 | 17 |
|  | EcxA metaloprotease toxin (*E. coli*) | 4L6T | 16-45 | 22.1 | 36 |
|  | E3 ubiquitin-protein ligase CBL (*H. sapines*) | 3BUX | 178-217 | 17.6 | 26 |

^a^See reference (Kelley et al., 2015) for details.

^b^Aligned residues. Part of protein sequence that is aligned with the template sequence.

^c^Confidence represents the probability (from 0 to 100) that the match between the query sequence and the corresponding template is a true homology. A match with confidence >90%, generally should indicate that the query sequence adopts the overall fold shown by the template and that the core of the protein is modelled at high accuracy.
