# Supplementary material for: Pirating conserved phage mechanisms promotes promiscuous staphylococcal pathogenicity island transfer
Source: eLife. 2017 Aug 8;6:e26487. doi: 10.7554/eLife.26487 (PMC5779228; doi:10.7554/eLife.26487)
Supplement: Supplementary file 5. — (B) Description and relationships between the PICI-encoded Stl repressorsa. [file elife-26487-supp5.docx]

**Supplementary file 5A. Phage-inducible chromosomal islands analysed in this study.**

| **PICI** | **Strain** | **Stl type** | **Accession number**  **(Stl protein)** |
| --- | --- | --- | --- |
| SaPIbov1 | S. aureus RF122 | SaPIbov1 | AAG29617 |
| SaPIC0673 | S. aureus C0673 | SaPIbov1 | EZX20759 |
| ShoCI794_SEPI | *S. hominis* 794_SEPI | SaPIbov1 | WP_049379208 |
| ShaCI51-48 | *S. haemolyticus* 51-48 | SaPIbov1 | WP_053031052 |
| SlCIVISLISI_25 | *S. lugdunensis* VISLISI_25 | SaPIbov1 | ARJ14897 |
| SsaCI3201 | *S. saprophyticus* 320*1* | SaPIbov1 | OEK45527 |
| SsiCIFDAARGOS_124 | *S. simulans* FDAARGOS_124 | SaPIbov1 | AMG96720 |
| SaPI2 | *S. aureus* RN3984 | SaPI2 | ABJ97305 |
| SarCISJTUF21285 | *S. argenteus* SJTU F21285 | SaPI2 | OMH99967 |
| ScCIM23864:W1 | *S. caprae* M23864:W1 | SaPI2 | EES42122 |
| SlCIFDAARGOS_141 | *S. lugdunensis* FDAARGOS_141 | SaPI2 | AMG62542 |
| ShaCI137133 | *S. haemolyticus* 137133 | SaPI2 | WP_053036598 |
| SeCINIHLM095 | *S. epidermidis* NIHLM095 | SaPI2 | EJD77777 |
| SsiCIUMC-CNS-990 | *S. simulans* UMC-CNS-990 | SaPI2 | WP_023015903 |
| SxCINS341 | *S. xylosus* NS341 | SaPI2 | KTW21949 |
| ScCIMF1871 | *S. capitis* MF1871 | SaPI2 | WP_019236000 |
| BdCIDSM17725 | *Bacillus decisifrondis* DSM 17725 | SaPI2 | WP_053595256 |
| SpyCIMGAS10750 | *Streptococcus pyogenes* MGAS10750 | SaPI2 | ABF38849 |

**Supplementary file 5B. Description and relationships between the PICI-encoded Stl repressors^a^.**

| **SaPIbov1-like repressors** |  |  |  |  |  |  |  |
| --- | --- | --- | --- | --- | --- | --- | --- |
| **PICI (Stl)** | **SaPIbov1** | **SaPIC0673** | **ShoCI1** | **ShaCI1** | **SlCIVISLISI_25** | **SsaCI3201** | **SsiCIFDAARGOS_124** |
| **SaPIbov1** | 100 | 49 | 45 | 41 | 99 | 79 | 45 |
| **SaPIC0673** | 49 | 100 | 49 | 42 | 49 | 48 | 50 |
| **ShoCI1** | 45 | 49 | 100 | 50 | 45 | 48 | 46 |
| **ShaCI1** | 41 | 42 | 50 | 100 | 40 | 43 | 44 |
| **SlCIVISLISI_25** | 99 | 49 | 45 | 40 | 100 | 78 | 45 |
| **SsaCI3201** | 79 | 48 | 48 | 43 | 78 | 100 | 45 |
| **SsiCIFDAARGOS_124** | 45 | 50 | 46 | 44 | 45 | 45 | 100 |

| **SaPI2-like repressors** |  |  |  |  |  |  |  |  |  |
| --- | --- | --- | --- | --- | --- | --- | --- | --- | --- |
| **PICI (Stl)** | **SaPI2** | **SarCI**  **SJTUF21285** | **ScCI**  **M23864:W1** | **SlCI**  **FDAARGOS_141** | **ShaCI2** | **SeCI**  **NIHLM095** | **SsiCI**  **UMC-CNS990** | **SxCI**  **NS341** | **ScCI**  **MF1871** |
| **SaPI2** | 100 | 100 | 99 | 99 | 50 | 46 | 58 | 50 | 47 |
| **SarCISJTUF21285** | 100 | 100 | 99 | 99 | 50 | 46 | 58 | 50 | 47 |
| **ScCIM23864:W1** | 99 | 99 | 100 | 100 | 50 | 46 | 58 | 50 | 47 |
| **SlCIFDAARGOS_141** | 99 | 99 | 100 | 100 | 50 | 46 | 58 | 50 | 47 |
| **ShaCI2** | 50 | 50 | 50 | 50 | 100 | 44 | 54 | 68 | 50 |
| **SeCINIHLM095** | 46 | 46 | 46 | 46 | 44 | 100 | 44 | 46 | 47 |
| **SsiCIUMC-CNS990** | 58 | 58 | 58 | 58 | 54 | 44 | 100 | 55 | 50 |
| **SxCINS341** | 50 | 50 | 50 | 50 | 68 | 46 | 55 | 100 | 50 |
| **ScCIMF1871** | 47 | 47 | 47 | 47 | 50 | 47 | 50 | 50 | 100 |

^a^Stl identities were determined with BLASTP. Shading: dark grey, >95 % identity to corresponding Stl protein from the PICI element described in the left column; light grey, 45–95 %; white, <45 %.
