# Supplementary material for: Pirating conserved phage mechanisms promotes promiscuous staphylococcal pathogenicity island transfer
Source: eLife. 2017 Aug 8;6:e26487. doi: 10.7554/eLife.26487 (PMC5779228; doi:10.7554/eLife.26487)
Supplement: Supplementary file 6. [file elife-26487-supp6.docx]

**Supplementary file 6. Putative SaPI inducers are present in phages infecting species other than *S. aureus*.**

| **SSAP family** | **Species** | **Identity (%)^a^** | **Accession number** |
| --- | --- | --- | --- |
| Sak | *Streptococcus equi subsp. equi* | 68 | CRV22907 |
|  | *Staphylococcus argenteus* | 100 | SGW93320 |
|  | *Staphylococcus pasteuri* | 87 | WP_072291905 |
|  | *Staphylococcus hominis* | 86 | WP_061544511 |
|  | *Staphylococcus capitis* | 86 | WP_047796056 |
|  | *Staphylococcus epidermidis* | 85 | YP_950708 |
|  | *Staphylococcus epidermidis* | 85 | YP_009302064 |
|  | *Staphylococcus epidermidis* | 85 | YP_950640 |
|  | *Staphylococcus saprophyticus* | 85 | WP_069796344 |
|  | *Staphylococcus equorum* | 82 | WP_046465215 |
|  | *Staphylococcus pettenkoferi* | 77 | WP_049408451 |
|  | *Staphylococcus succinus* | 75 | WP_063410298 |
|  | *Staphylococcus gallinarum* | 73 | WP_042738301 |
|  | *Staphylococcus cohnii* | 71 | WP_040030394 |
|  | *Staphylococcus xylosus* | 70 | WP_039067702 |
|  | *Staphylococcus haemolyticus* | 67 | WP_053037817 |
| Sak4 | *Streptococcus pneumoniae* | 88 | WP_061749171 |
|  | *Staphylococcus epidermidis* | 98 | WP_002486391 |
|  | *Staphylococcus capitis* | 88 | WP_064210320 |
|  | *Staphylococcus xylosus* | 88 | WP_069795761 |
|  | *Staphylococcus equorum* | 85 | OEK70855 |
| Erf | *Streptococcus pneumoniae* | 60 | CVY54899 |
|  | *Listeria monocytogenes* | 70 | WP_034172821 |
|  | *Streptococcus equi subsp. equi* | 68 | CRV30450 |
|  | *Staphylococcus argenteus* | 99 | SGX09933 |
|  | *Staphylococcus epidermidis* | 83 | WP_002495407 |
|  | *Staphylococcus equorum* | 78 | WP_069813043 |
|  | *Staphylococcus pasteuri* | 76 | KKI54766.1 |
|  | *Staphylococcus warneri* | 75 | WP_049423834 |
|  | *Staphylococcus haemolyticus* | 71 | WP_053018506 |
|  | *Staphylococcus xylosus* | 70 | WP_047172550 |
|  | *Staphylococcus capitis* | 70 | WP_002469948 |
|  | *Staphylococcus lugdunensis* N920143 | 69 | CCB53297 |
|  | *Staphylococcus carnosus* | 69 | WP_046100578 |
|  | *Staphylococcus hominis* | 68 | WP_071859763 |
|  | *Staphylococcus agnetis* | 68 | ALN76777 |
|  | *Staphylococcus simulans* | 56 | WP_023015642 |
| Redβ | *Streptococcus agalactiae* | 40 | WP_017827951 |
|  | *Streptococcus suis* | 38 | WP_044775177 |
|  | *Staphylococcus epidermidis* | 83 | WP_063280508 |
|  | *Staphylococcus epidermidis* | 83 | CUY00565 |
|  | *Staphylococcus epidermidis* | 51 | OAW38008 |

^a^At protein level. The different proteins were compared with the prototypical members of each family: Sak: ORF16 80α; Sak4: ORF16 ɸ52A; Erf: ORF17 ɸSLT; and Redβ: SA1794 ɸN315.

| **Dut** | **Species** | **Identity (%)^a^** | **Accession number** |
| --- | --- | --- | --- |
| Dimeric φDI | *Staphylococcus schweitzeri* | 93 | CDR62164 |
|  | *Staphylococcus hominis* | 83 | WP_049431351 |
|  | *Staphylococcus capitis* | 82 | WP_072098691 |
|  | *Staphylococcus saprophyticus* | 79 | WP_069878160 |
|  | *Staphylococcus argenteus* | 71 | WP_049283901 |
| Trimeric φ80α | *Staphylococcus argenteus* | 79 | WP_072599576 |
|  | *Staphylococcus intermedius* | 76 | WP_019168106 |
|  | *Staphylococcus chromogenes* | 73 | WP_037572024 |

^a^At protein level. The different proteins were compared with the prototypical members of each family: Dimeric: φDI Dut; Trimeric: 80α Dut.
