# Supplementary material for: Pirating conserved phage mechanisms promotes promiscuous staphylococcal pathogenicity island transfer
Source: eLife. 2017 Aug 8;6:e26487. doi: 10.7554/eLife.26487 (PMC5779228; doi:10.7554/eLife.26487)
Supplement: Supplementary file 7. [file elife-26487-supp7.docx]

**Supplementary file 7. Inter-species PICI transfer.**

| **PICI** | **Species** | **Accession number**  **(Genomic location)** | **Size**  **(nt)** | ***att* site core** | **Accessory genes** |
| --- | --- | --- | --- | --- | --- |
| ScCIM23864:W1 | *S. caprae* | ACJB01000009  (5416-19262) | 13847 | atgccaggtatgatgtaa | FeuA (transport of metal ions);  YktD ( polyketide biosynthesis) |
| SlCIFDAARGOS_141 | *S. lugdunensis* | CP014022  (2448810-2462656) | 13847 | atgccaggtatgatgtaa | FeuA; YktD |
