# Supplementary material for: Pirating conserved phage mechanisms promotes promiscuous staphylococcal pathogenicity island transfer
Source: eLife. 2017 Aug 8;6:e26487. doi: 10.7554/eLife.26487 (PMC5779228; doi:10.7554/eLife.26487)
Supplement: Supplementary file 8. — (B) Plasmids used in this study. (C) Oligonucleotide designs used in this study. [file elife-26487-supp8.docx]

**Supplementary file 8A. Bacterial strains used in this study.**

| **Strains** | **Description** | **Reference** |
| --- | --- | --- |
| DH5α | Host for DNA cloning |  |
| RN4220 | Restriction-defective derivate of RN450 | (Kreiswirth et al., 1983) |
| RN450 | NCTC8325 cured of φ11, φ12 and φ13 | (Novick, 1967) |
| BL21 (DE3) | *E. coli* expression strain | Stratagene |
| JP6774 | RN4220 Δ*spa* SaPIbov1 *tst*::*tet*M | (Tormo-Más et al., 2010) |
| JP11634 | RN4220 SaPIbov5::*erm*C adjusted | (Carpena et al., 2016) |
| JP12491 | RN4220 pJP674 | (Tormo-Más et al., 2010) |
| JP5468 | RN4220 pJP674 pJP653 | (Tormo-Más et al., 2010) |
| RN10539 | RN450 lysogenic for 80α | (Ubeda et al., 2007) |
| JP12884 | Lysate of φ80 | (Christie et al., 2010) |
| JP15166 | BL21 DE3 pJP1987 | This work |
| JP15167 | BL21 DE3 pJP1988 | This work |
| JP13792 | BTH101: BACTH System Kit, Bacterial Adenylate Cyclase Two-hybrid System Kit | EUROMEDEX |
| JP975 | *S. aureus* N315 | (Kuroda et al., 2001) |
| JP5011 | RN4220 lysogenic for φSLT *pvl*::*tet*M | (Ferrer et al., 2011) |
| JP2878 | RN4220 SaPI2 *tst*::*tet*M | (Subedi et al., 2007) |
| JP15847 | RN4220 Δ*spa* SaPI2 *tst*::*tet*M | This work |
| JP14818 | RN4220 pJP674 pJP1928 | This work |
| JP13056 | RN4220 pJP674 pJP1927 | This work |
| JP14835 | RN4220 pJP674 pJP1930 | This work |
| JP15091 | RN4220 pJP1925 | This work |
| JP15092 | RN4220 pJP1925 JP1928 | This work |
| JP15093 | RN4220 pJP1925 pJP1927 | This work |
| JP15095 | RN4220 pJP1925 pJP1930 | This work |
| JP15097 | RN4220 pJP1925 pJP653 | This work |
| JP15098 | RN4220 pJP1926 | This work |
| JP15099 | RN4220 pJP1926 JP1928 | This work |
| JP15100 | RN4220 pJP1926 pJP1927 | This work |
| JP15102 | RN4220 pJP1926 pJP1930 | This work |
| JP15104 | RN4220 pJP1926 pJP653 | This work |
| JP15105 | RN4220 pJP674 pCN51 | This work |
| JP15106 | RN4220 pJP1924 pCN51 | This work |
| JP15107 | RN4220 pJP1925 pCN51 | This work |
| JP15108 | RN4220 pJP1926 pCN51 | This work |
| JP14832 | BL21 DE(3) pJP1932 | This work |
| JP14833 | BL21 DE(3) pJP1933 | This work |
| JP10560 | BL21 DE(3) pJP1938 | This work |
| JP14649 | JP6774 pJP1928 | This work |
| JP14817 | JP11634 pJP1931 | This work |
| JP15161 | RN4220 + 80α chimera ORF16-17 | This work |
| JP2592 | RN4220 φ52A | Lab strain |

| **Strains** | **Description** | **Reference** |
| --- | --- | --- |
| JP4729 | *S. epidermidis* φPH15 | Lab strain |
| JP15927 | JP15847 pJP2050 | This work |
| JP15928 | JP15847 pJP2051 | This work |
| JP15929 | JP15847 pJP2052 | This work |
| JP15930 | JP15847 pJP2053 | This work |
| JP15932 | JP15847 pJP2054 | This work |
| JP15933 | JP15847 pCN51 | This work |
| JP13969 | RN4220 pJP1977 pJP1883 | This work |
| JP13970 | RN4220 pJP1977 pJP1965 | This work |
| JP13971 | RN4220 pJP1977 pJP1966 | This work |
| JP14737 | RN4220 pJP1977 pJP1967 | This work |
| JP15164 | RN4220 pJP1977 pJP1968 | This work |
| JP13975 | RN4220 pJP1977 pJP1969 | This work |
| JP13976 | RN4220 pJP1977 pCN51 | This work |
| JP13927 | RN4220 pJP1978 pJP1883 | This work |
| JP13929 | RN4220 pJP1978 pJP1965 | This work |
| JP13931 | RN4220 pJP1978 pJP1966 | This work |
| JP14738 | RN4220 pJP1978 pJP1967 | This work |
| JP13932 | RN4220 pJP1978 pJP1968 | This work |
| JP13936 | RN4220 pJP1978 pJP1969 | This work |
| JP13937 | RN4220 pJP1978 pCN51 | This work |
| JP13938 | RN4220 pJP1980 pJP1883 | This work |
| JP13940 | RN4220 pJP1980 pJP1965 | This work |
| JP13942 | RN4220 pJP1980 pJP1966 | This work |
| JP14740 | RN4220 pJP1980 pJP1967 | This work |
| JP13943 | RN4220 pJP1980 pJP1968 | This work |
| JP13947 | RN4220 pJP1980 JP1969 | This work |
| JP13948 | RN4220 pJP1980 pCN51 | This work |
| JP13977 | RN4220 pJP1979 pJP1883 | This work |
| JP13978 | RN4220 pJP1979 pJP1965 | This work |
| JP13979 | RN4220 pJP1979 pJP1966 | This work |
| JP14739 | RN4220 pJP1979 pJP1967 | This work |
| JP15165 | RN4220 pJP1979 pJP1968 | This work |
| JP13983 | RN4220 pJP1979 pJP1969 | This work |
| JP13984 | RN4220 pJP1979 pCN51 | This work |
| JP15168 | BTH101 pJP1986 pJP1981 | This work |
| JP15169 | BTH101 pJP1986 pJP1982 | This work |
| JP15170 | BTH101 pJP1986 pJP1983 | This work |
| JP15171 | BTH101 pJP1986 pJP1984 | This work |
| JP15172 | BTH101 pJP1986 pJP1985 | This work |
| JP15173 | BTH101 pUT18c pKNT25 | This work |
| JP15174 | BTH101 pUT18c-Zip pKT25-Zip | This work |

**Supplementary file 8B. Plasmids used in this study.**

| **Plasmid** | **Description** | **Reference** |
| --- | --- | --- |
| pPROEX HTa | Expression vector | Invitrogen |
| pCU1 | Expression vector | (Augustin et al., 1992) |
| pCN36 | Expression vector | (Charpentier et al., 2004) |
| pCN41 | Expression vector | (Charpentier et al., 2004) |
| pCN51 | Expression vector | (Charpentier et al., 2004) |
| pJP674 | pRN8298-chlor-pInt-20-19-18-*bla*Z (SaPIbov1) | (Tormo-Más et al., 2010) |
| pUT18c | Two hybrid assay plasmid | EUROMEDEX |
| pKNT25 | Two hybrid assay plasmid | EUROMEDEX |
| pUT18c-Zip | Two hybrid assay plasmid | EUROMEDEX |
| pKT25-Zip | Two hybrid assay plasmid | EUROMEDEX |
| pJP653 | pCN51-3xflag-φ11 Dut | (Tormo-Más et al., 2010) |
| pJP1969 | pCN51 80α ORF16-17 chimera | This work |
| pJP1987 | pPROEX HTa His-Stl_SaPI2_ + ORF16 of 80α | This work |
| pJP1988 | pPROEX HTa His-Stl_SaPI2_ + ORF16-17 chimera 80α | This work |
| pJP1925 | pRN8298-chlor-*stl*-*str*-*xis-bla*Z (ShoCI794_SEPI) | This work |
| pJP1926 | pRN8298-chlor-*stl*-*str*-*xis-bla*Z (ShaCI51-48) | This work |
| pJP1928 | pCN51-3xflag-O11 Dut | This work |
| pJP1927 | pCN51-3xflag-NM1 Dut | This work |
| pJP1930 | pCN51-3xflag-IPLA6 Dut | This work |
| pJP1931 | pCN51-3tet-3xflag-O11 Dut | This work |
| pJP1932 | pPROEX HTa His-Stl_SaPIbov1_ + Dut_ΦO11_ | This work |
| pJP1933 | pPROEX HTa Stl_SaPIbov1_ + Dut_ΦO11_ | This work |
| pJP1938 | pET28a-*dut* φO11 | This work |
| pJP1883 | pCN51 80α ORF16 | (Neamah et al., 2017) |
| pJP1965 | pCN51 Φ52A ORF16 | This work |
| pJP1966 | pCN51 ORF17 ΦSLT | This work |
| pJP1967 | pCN51 ΦN315 ORFRS1794 | This work |
| pJP1968 | pCN51 Φ15 ORF46 (*S. epidermidis*) | This work |
| pJP1970 | pCN41 SaPI2 *stl-str* region | This work |
| pJP2050 | pCN51-3xflag-80α ORF16 | This work |
| pJP2051 | pCN51-3xflag-φ52A ORF16 | This work |
| pJP2052 | pCN51-3xflag-φSLT ORF17 | This work |
| pJP2053 | pCN51-3xflag-φN315 ORFRS1794 | This work |
| pJP2054 | pCN51-3xflag- 80α Chimeric 16-17 | This work |
| pJP1971 | pCU57 SaPI2-like *stl-str* region of SsiCIUMC-CNS990 (*S. simulans*) | This work |

| **Plasmid** | **Description** | **Reference** |
| --- | --- | --- |
| pJP1972 | pCU57 SaPI2-like *stl-str* region of SeCINIHLM095 (*S. epidermidis*) | This work |
| pJP1973 | pCU57 SaPI2-like *stl*-*str* region of ShaCI137133 (*S. haemolyticus*) | This work |
| pJP1974 | pCN41 SaPI2-like *stl-str* region of SeCINIHLM095 | This work |
| pJP1975 | pCN41 SaPI2-like *stl-str* region of ShaCI137133 | This work |
| pJP1976 | pCN41 SaPI2-like *stl-str* region of ScCIUMC-CNS990 | This work |
| pJP1977 | pCU1 SaPI2 *stl-str* region + *bla*Z | This work |
| pJP1978 | pCU1 SaPI2-like *stl-str* region of SeCINIHLM095 + *bla*Z | This work |
| pJP1979 | pCU1 SaPI2-like *stl-str* region of ShaCI137133 + *bla*Z | This work |
| pJP1980 | pCU1 SaPI2-like *stl-str* region of SsiCIUMC-CNS990 + *bla*Z | This work |
| pJP1981 | pUT18c 80α ORF16 | This work |
| pJP1982 | pUT18c φ52A ORF16 | This work |
| pJP1983 | pUT18c φSLT ORF17 | This work |
| pJP1984 | pUT18c φN315 ORFRS1794 | This work |
| pJP1985 | pUT18c φ80a ORF16-17 chimera | This work |
| pJP1986 | pKNT25 *stl*_SaPI2 | This work |

**Supplementary file 8C. Oligonucleotide designs used in this study.**

| **Plasmid** | **Oligonucleotides** | **Sequence** |  |
| --- | --- | --- | --- |
| pJP674 | SaPIbov1-149cB  NY-24mK | CGCGGATCCGATCAGTACCTAAATATGCG  CGGGGTACCCACTCGGTTATAACCTT |  |
| pJP1925 | Sepid-Stl-1mB  Sepid-Stl-2cK | CGCGGATCCGGAGAAGTTATTTTGAATTTTTGATTTGTTC  CGGGGTACCCTTTTGCTGGGCGATTTGCTCTGCAAGTTTC |  |
| pJP1926 | Shaem-Stl-1mB  Shaem-Stl-2cK | CGCGGATCCGCGAGAAAAGTCATTTACAATTTTTAAATTG  CGGGGTACCCGCTTCTGTTGTTGTTCTATCTTTTGCTGTG |  |
| pJP1928  pJP1927 | dutNM1-1mS  dutNM1-2m  dut-DI-2cB | ACGCGTCGACATTATGACGGGTCAAGTTGTCTATAAATATGAGGAGGCACAGGAAAATGGATTATAAAGATCACGATGGCGATTATAAAGATC  CACGATGGCGATTATAAAGATCACGATATCGATTATAAAGATGATGATGATAAAATGACTAACACATTAACAATTGATCAG  CGCGGATCCTTACACGTATCCTTTTCCTGC  (These plasmids were constructed using the same primers with different templates.) |  |
| pJP1930 | dutNM1-1mS  IPLA5_0063_2m  IPLA5_0063_3cB | ACGCGTCGACATTATGACGGGTCAAGTTGTCTATAAATATGAGGAGGCACAGGAAAATGGATTATAAAGATCACGATGGCGATTATAAAGATC  CGATGGCGATTATAAAGATCACGATATCGATTATAAAGATGATGATGATAAAATGAGTAAGAAATTAGAAATTAAATTG  CGCGGATCCTTAGAATCCTGTTGATCCAAACCC |  |
| pJP1931 | dutNM1-1mS  dutNM1-2m  dut-DI-2cB | ACGCGTCGACATTATGACGGGTCAAGTTGTCTATAAATATGAGGAGGCACAGGAAAATGGATTATAAAGATCACGATGGCGATTATAAAGATC  CACGATGGCGATTATAAAGATCACGATATCGATTATAAAGATGATGATGATAAAATGACTAACACATTAACAATTGATCAG  CGCGGATCCTTACACGTATCCTTTTCCTGC  (Tetracycline marker from pCN36 used to replace the Erythromycin marker in pCN51) | |

| **Plasmid** | **Oligonucleotides** | **Sequence** |
| --- | --- | --- |
| pJP1932  pJP1933 | Stl_SaPIbov1_  SaPIbov1- 203 mB  SaPIbov1-223cE  dUTPase_ΦO11_  dutNM1-35mS  phiO11_Dut_1cP | CGCGGATCCCATGGAAGGAGCTGGTCAAATGGC  CCGGAATTCGATTAATTAGTGTCTTTTTCAAG  ACGCGTCGACTTCACACAGGAAACAGACCATGACTAACACATTAACAATTG  CTGCAGTTACACGTATCCTTTTCCTGCG  (These plasmids were constructed using the same primers with different templates. pJP1933 was created by using Klenow to transfer the His-tag to a different reading frame from the Stl_SaPIbov1_.) |
| pJP1938 | dutNM1-19cS  dutNM1-18mB | ACGCGTCGACTTACACGTATCCTTTTCCTGC CGCGGATCCATGACTAACACATTAACAATTGATC |
| pJP1965 | ORF13 phi80-1mS  ORF13 phi80-1cB | ACGCGTCGACCGGTAAAGGTGGGAGAATAG  CGCGGATCCGTGATTTTCATAATTTTTATAC |
| pJP1966 | ORF17 phiSLT-1mS  ORF17 phiSLT-1cB | ACGCGTCGACGAGGGTGCTAGCATTGTTGAG  CGCGGATCCCCTTTTAACTCTGGATCTGC |
| pJP1967 | Rec phiN315-1mB  Rec phiN315-2cE | CGCGGATCCCAAAACAGAATCACAACAAATTC  CCGGAATTCGTAGCAGTTACCACTTGAACC |
| pJP1968 | ORF46 phi15 S.epi-1mS  ORF46 phi15 S.epi-1cB | ACGCGTCGACCTAAGCACAGAGCAATTAAAAAG  CGCGGATCCCTAAATCTTTAGTGATACGTCC |
| pJP1969 | ORF16 phi80α-21mS  ORF16-17 chi phi80α 6cB | ACGCGTCGACTGATATGTCTAAGCACAAAGC  CGCGGATCCTCAGAACGGTAAGTCATCATC |
| pJP1970 | Stl SaPI2-1mS  Str SaPI2-1cB | ACGCGTCGACTTAATATTCTTTAAAAATATCACTAGATAAACGGC  CGCGGATCCTTATGCCTCCTTTACTTCAAATTC |
| pJP1977 | Stl SaPI2-1mS  blaZ-1cHindIII | ACGCGTCGACTTAATATTCTTTAAAAATATCACTAGATAAACGGC  CCCAAGCTTGGGGTTATCAGTATTTATTATGCATTTAG |
| pJP1978 | Stl-Se-1mS  blaZ-1cHindIII | ACGCGTCGACGGCATGCCTGCAGGTCGACCCTAC  CCCAAGCTTGGGGTTATCAGTATTTATTATGCATTTAG |
| pJP1980 | Stl-Se-1mS  blaZ-1cHindIII | ACGCGTCGACGGCATGCCTGCAGGTCGACCCTAC  CCCAAGCTTGGGGTTATCAGTATTTATTATGCATTTAG |
| pJP1979 | Stl-Sh-2m  blaZ-2cSphI | CTGCAGGTCGACCCTACTTAATATTC  ACATGCATGCGATGTTATCAGTATTTATTATG |
| pJP1981 | T-ORF16 phi80α-4mS  T-ORF16 phi80α-5cB | ACGCGTCGACAATGACTGAACAAACATTATTTG  CGCGGATCCTTATTGTTTCTCCTCACTATC |
| **Plasmid** | **Oligonucleotides** | **Sequence** |
| pJP1982 | T-ORF16 phi52A-5mS  T-ORF16 phi52A-6cB | ACGCGTCGACAATGACTGAAAAAACTAATCAAG  CGCGGATCCTTAATTACCATTTCTAATTGC |
| pJP1983 | T-ORF17 phiSLT-6mS  T-ORF17 phiSLT-6cB | ACGCGTCGACAATGGCCGAACAACTTAATTTG  CGCGGATCCTCATTGTTCAATTCCTCCAAG |
| pJP1984 | T-rec phiN315 6mS  T-rec phiN315 6cB | ACGCGTCGACAATGACGAATGAATTACTATTAAAAAAC  CGCGGATCCTTAAAATGGCTCTTCTTCGCTTTC |
| pJP1985 | T-ORF16 phi80α-4mS  ORFchi phi80α-6cB | ACGCGTCGACAATGACTGAACAAACATTATTTG  CGCGGATCCTCAGAACGGTAAGTCATCATC |
| pJP1986 | TN-stl_SaPI2-6mB  TN-stl_SaPI2-6cKpnI | CGCGGATCCCATGATTAGAAATAGATTGTCTG  GGGGTACCATATATTCTTTAAAAATATCACTAGATAAACGGCTATC |
| pJP1987 | SaPI2-12mB  SaPI2-13cE  orf16-phi80α-15mS  orf16-phi80α-16mXb | CGCGGATCCGATGATTAGAAATAGATTGTCTG  CCGGAATTCTGATCACCTCGATTAATATTC  ACGCGTCGACTAACAATTTCACACAGGAAACAGACCATGACTGAACAAACATTATTTG  GCTCTAGATTATTGTTTCTCCTCACTATCC |
| pJP1988 | SaPI2-12mB  SaPI2-13cE  ORF16-17 chi phi80α-8mS  ORF16-17 chi phi80α-9cXbaI | CGCGGATCCGATGATTAGAAATAGATTGTCTG  CCGGAATTCTGATCACCTCGATTAATATTC  ACGCGTCGACTAACAATTTCACACAGGAAACAGACCATGACTGAACAAACATTATTTG  GCTCTAGATCAGAACGGTAAGTCATCATC |
| pJP2050 | ORF16 phi80α _flag-6mS  Orf16-phi80α-13m  Orf16phi80α-20cB | ACGCGTCGACTGATATGTCTAAGCACAAAGCAATCAAGAAAACAGTGACAGAAACTATTGAGTACGAGGAGGTAGAACATGGATTATAAAGATCACGATGG  ATGGATTATAAAGATCACGATGGCGATTATAAAGATCACGATATCGATTATAAAGATGATGATGATAAAATGACTGAACAAACATTATTTG  CGCGGATCCTTATTGTTTCTCCTCACTATC |
| pJP2051 | ORF16 phi80α_flag-6mS  ORF16 phi52A-17m  ORF13 phi80-1cB | ACGCGTCGACTGATATGTCTAAGCACAAAGCAATCAAGAAAACAGTGACAGAAACTATTGAGTACGAGGAGGTAGAACATGGATTATAAAGATCACGATGG  ATGGATTATAAAGATCACGATGGCGATTATAAAGATCACGATATCGATTATAAAGATGATGATGATAAAATGACTGAAAAAACTAATCAAGATG  CGCGGATCCGTGATTTTCATAATTTTTATAC |

| **Plasmid** | **Oligonucleotides** | **Sequence** |
| --- | --- | --- |
| pJP2052 | ORF16 phi80α_flag-6mS  ORF17 phiSLT-17m  ORF17 phiSLT-1cB | ACGCGTCGACTGATATGTCTAAGCACAAAGCAATCAAGAAAACAGTGACAGAAACTATTGAGTACGAGGAGGTAGAACATGGATTATAAAGATCACGATGG  ATGGATTATAAAGATCACGATGGCGATTATAAAGATCACGATATCGATTATAAAGATGATGATGATAAAATGGCCGAACAACTTAATTTG  CGCGGATCCCCTTTTAACTCTGGATCTGC |
| pJP2053 | ORF16 phi80α_flag-6mS  ORF10305 phiN315-17m  Rec phiN315-2cE | ACGCGTCGACTGATATGTCTAAGCACAAAGCAATCAAGAAAACAGTGACAGAAACTATTGAGTACGAGGAGGTAGAACATGGATTATAAAGATCACGATGG  ATGGATTATAAAGATCACGATGGCGATTATAAAGATCACGATATCGATTATAAAGATGATGATGATAAAATGACGAATGAATTACTATTAAAAAACA  CCGGAATTCGTAGCAGTTACCACTTGAACC |
| pJP5054 | ORF16 phi80α_flag-6mS  orf16-phi80a-13m  orf17phi80a-10cB | ACGCGTCGACTGATATGTCTAAGCACAAAGCAATCAAGAAAACAGTGACAGAAACTATTGAGTACGAGGAGGTAGAACATGGATTATAAAGATCACGATGG  ATGGATTATAAAGATCACGATGGCGATTATAAAGATCACGATATCGATTATAAAGATGATGATGATAAAATGACTGAACAAACATTATTTG  CGCGGATCCTCAGAACGGTAAGTCATCATC |
| pETNKI-StlΔ^HTH^ | Stl_M1-K176_Fw | CGATAAAAAATAAGAAGTAACAATAGAAGAAATTG |
|  | Stl_M1-K176_Rv | CTTGATTTTCGAGTATTTGCTAAAG |
| pETNKI-StlΔ^Cter^ | Stl_T87-N267_Fw | CCAGCAGCAGACGGGAGGTATGACATTAAA |
|  | Stl_T87-N267_Rv | GGCGGCGGAGCCCGTTAATTAGTGTCTTTT |
| Sequences recognized by the restriction enzymes used in cloning are underlined. | | |

| **Probe** | **Oligonucleotides** | **Sequence** |
| --- | --- | --- |
| **SaPIbov1/SaPIbov5** | SaPIbov1-112mE SaPIbov1-113cB | CCGGAATTCAATTGCTGAGGCAAAACTTC  CGCGGATCCTAATTCTCCACGTCTAAAGC |
| **SaPI2** | Tet-1m  Tet-2c | GTGGACAAAGGTACAACGAGG  CTTTCCTCTTGTTCGAGTTCC |

**References**

Augustin, J., Rosenstein, R., Wieland, B., Schneider, U., Schnell, N., Engelke, G., et al. (1992). Genetic analysis of epidermin biosynthetic genes and epidermin-negative mutants of Staphylococcus epidermidis. *European Journal of Biochemistry / FEBS*, *204*(3), 1149–1154.

Carpena, N., Manning, K. A., Dokland, T., Marina, A., & Penadés, J. R. (2016). Convergent evolution of pathogenicity islands in helper cos phage interference. *Philosophical Transactions of the Royal Society of London. Series B, Biological Sciences*, *371*(1707), 20150505. http://doi.org/10.1098/rstb.2015.0505

Charpentier, E., Anton, A. I., Barry, P., Alfonso, B., Fang, Y., & Novick, R. P. (2004). Novel cassette-based shuttle vector system for gram-positive bacteria. *Applied and Environmental Microbiology*, *70*(10), 6076–6085. http://doi.org/10.1128/AEM.70.10.6076-6085.2004

Christie, G. E., Matthews, A. M., King, D. G., Lane, K. D., Olivarez, N. P., Tallent, S. M., et al. (2010). The complete genomes of Staphylococcus aureus bacteriophages 80 and 80α--implications for the specificity of SaPI mobilization. *Virology*, *407*(2), 381–390. http://doi.org/10.1016/j.virol.2010.08.036

Ferrer, M. D., Quiles-Puchalt, N., Harwich, M. D., Tormo-Más, M. Á., Campoy, S., Barbé, J., et al. (2011). RinA controls phage-mediated packaging and transfer of virulence genes in Gram-positive bacteria. *Nucleic Acids Research*, *39*(14), 5866–5878. http://doi.org/10.1093/nar/gkr158

Kelley, L. A., Mezulis, S., Yates, C. M., Wass, M. N., & Sternberg, M. J. E. (2015). The Phyre2 web portal for protein modeling, prediction and analysis. *Nature Protocols*, *10*(6), 845–858. http://doi.org/10.1038/nprot.2015.053

Kreiswirth, B. N., Löfdahl, S., Betley, M. J., O'Reilly, M., Schlievert, P. M., Bergdoll, M. S., & Novick, R. P. (1983). The toxic shock syndrome exotoxin structural gene is not detectably transmitted by a prophage. *Nature*, *305*(5936), 709–712.

Kuroda, M., Ohta, T., Uchiyama, I., Baba, T., Yuzawa, H., Kobayashi, I., et al. (2001). Whole genome sequencing of meticillin-resistant Staphylococcus aureus. *Lancet*, *357*(9264), 1225–1240.

Neamah, M. M., Mir-Sanchis, I., López-Sanz, M., Acosta, S., Baquedano, I., Haag, A. F., et al. (2017). Sak and Sak4 recombinases are required for bacteriophage replication in Staphylococcus aureus. *Nucleic Acids Research*. http://doi.org/10.1093/nar/gkx308

Novick, R. (1967). Properties of a cryptic high-frequency transducing phage in Staphylococcus aureus. *Virology*, *33*(1), 155–166.

Subedi, A., Ubeda, C., Adhikari, R. P., Penadés, J. R., & Novick, R. P. (2007). Sequence analysis reveals genetic exchanges and intraspecific spread of SaPI2, a pathogenicity island involved in menstrual toxic shock. *Microbiology (Reading, England)*, *153*(Pt 10), 3235–3245. http://doi.org/10.1099/mic.0.2007/006932-0

Tormo-Más, M. Á., Mir, I., Shrestha, A., Tallent, S. M., Campoy, S., Lasa, I., et al. (2010). Moonlighting bacteriophage proteins derepress staphylococcal pathogenicity islands. *Nature*, *465*(7299), 779–782. http://doi.org/10.1038/nature09065

Ubeda, C., Barry, P., Penadés, J. R., & Novick, R. P. (2007). A pathogenicity island replicon in Staphylococcus aureus replicates as an unstable plasmid. *Proceedings of the National Academy of Sciences of the United States of America*, *104*(36), 14182–14188. http://doi.org/10.1073/pnas.0705994104

Yang, J., Yan, R., Roy, A., Xu, D., Poisson, J., & Zhang, Y. (2015). The I-TASSER Suite: protein structure and function prediction. *Nature Methods*, *12*(1), 7–8. http://doi.org/10.1038/nmeth.3213
